# Deep convolutional neural networks for classifying head and neck cancer using hyperspectral imaging

**DOI:** 10.1117/1.JBO.22.6.060503

**Published:** 2017-06-24

**Authors:** Martin Halicek, Guolan Lu, James V. Little, Xu Wang, Mihir Patel, Christopher C. Griffith, Mark W. El-Deiry, Amy Y. Chen, Baowei Fei

**Affiliations:** a Georgia Institute of Technology and Emory University, The Wallace H. Coulter Department of Biomedical Engineering, Atlanta, Georgia, United States; b Medical College of Georgia, Augusta, Georgia, United States; c Emory University School of Medicine, Department of Pathology and Laboratory Medicine, Atlanta, Georgia, United States; d Emory University School of Medicine, Department of Hematology and Medical Oncology, Atlanta, Georgia, United States; e Emory University School of Medicine, Department of Otolaryngology, Atlanta, Georgia, United States; f Winship Cancer Institute of Emory University, Atlanta, Georgia, United States; g Emory University, Department of Radiology and Imaging Sciences, Atlanta, Georgia, United States; h Emory University, Department of Mathematics and Computer Science, Atlanta, Georgia, United States

**Keywords:** hyperspectral imaging, convolutional neural network, cancer detection, deep learning, image-guided surgery

## Abstract

Surgical cancer resection requires an accurate and timely diagnosis of the cancer margins in order to achieve successful patient remission. Hyperspectral imaging (HSI) has emerged as a useful, noncontact technique for acquiring spectral and optical properties of tissue. A convolutional neural network (CNN) classifier is developed to classify excised, squamous-cell carcinoma, thyroid cancer, and normal head and neck tissue samples using HSI. The CNN classification was validated by the manual annotation of a pathologist specialized in head and neck cancer. The preliminary results of 50 patients indicate the potential of HSI and deep learning for automatic tissue-labeling of surgical specimens of head and neck patients.

## 1 Introduction

Hyperspectral imaging (HSI) is a noncontact imaging modality that acquires a two-dimensional image over discrete wavelengths, producing a hyperspectral image cube (hypercube). HSI has recently been promisingly used for biomedical imaging, despite its origin in geological remote sensing.^1^

Surgery remains the well-established, standard treatment for most cancers, including thyroid and oral cancer, which is the sixth most common cancer worldwide.^2^ Recurrence rates for cancer after surgical resection are largely dependent on negative (cancer-free) surgical margins, along with other factors that cannot be controlled, such as extremes in patient age and other patient demographics.^3^ Surgical cancer resection can be a lengthy procedure and sometimes involves “free-flap” reconstruction of the resected area with skin removed from a different part of the body, commonly the arm or the leg. In extreme cases, cancer of the thyroid can also become locally invasive requiring removal of the larynx or adjacent structures.^2^

Cancer-margin detection in head and neck cancer is essential for salvaging the valuable normal tissue needed to preserve as much patient function as possible. During these surgeries, surgeons require an accurate and timely diagnosis of malignant areas that require resection. A rapid and reliable surgical diagnostic aid that provides tissue and cancer identification would prove very efficacious in the surgical theater. Previously, regression-based machine learning algorithms, such as support vector machines (SVMs) and k-nearest neighbor (kNN), have been applied to HSI in attempt solve this problem.^1^

In this letter, a method for automated classification of normal and cancerous, head and neck tissue is developed using deep convolutional neural networks (CNNs). This work demonstrates that deep learning has the potential to be implemented into a tissue classifier, fully trainable on a database of hyperspectral images from tissue specimens that can produce near real-time tissue labeling for intraoperative cancer detection.

## 2 Materials and Methods

### 2.1 Study Design

For this study, we recruited 50 head and neck cancer patients who were undergoing surgical cancer resection in order to collect 88 excised tissue samples. We collaborated with the Emory University Hospital Midtown surgical and pathology teams to obtain three tissue samples from each patient, i.e., a sample of the tumor, a normal tissue sample, and a sample at the tumor–normal interface. After the tissues are resected, the samples are imaged with HSI in order to obtain the hypercube.

The average patient age was 57. The two origin sites included for cancer resection were upper aerodigestive tract sites, i.e., tongue, larynx, pharynx, mandible, and the thyroid. Of the 50 patients, 29 had squamous-cell carcinoma (SCCa) and 21 had thyroid carcinoma, i.e., papillary thyroid carcinoma and medullary thyroid carcinoma.

### 2.2 Hyperspectral Imaging

Hyperspectral images were acquired for all tissues samples from the 50 cancer patients using a CRI Maestro imaging system (Perkin Elmer Inc., Waltham, Massachusetts). The imaging system is comprised of a Xenon white-light illumination source, a liquid crystal tunable filter to separate spectral bands, and a 16-bit charge-coupled device capable of obtaining high-resolution images (1040×1392  pixels).^4^ The images were obtained over a spectral bandwidth from 450 to 900 nm in 5-nm increments, producing a hypercube with 91 spectral bands.

### 2.3 Image Preprocessing

Hyperspectral data normalization was performed in order to compare different patients’ samples and different cancer types. Each patient’s hypercube was normalized in order to obtain arbitrary units of reflectance by dividing the reflectance values by a white reference after subtracting the dark current.^5^ Then, a 3×3 median filter was applied to each band within the hypercube. Next, for each hypercube, all pixel intensities were binned in a histogram and a gamma distribution was fit to the binning distribution. A population threshold was determined experimentally to sufficiently remove most glare pixels by visual inspection, which corresponds to the top 0.05% to 0.2% of the pixel intensities.^5^

**Fig. 1:**
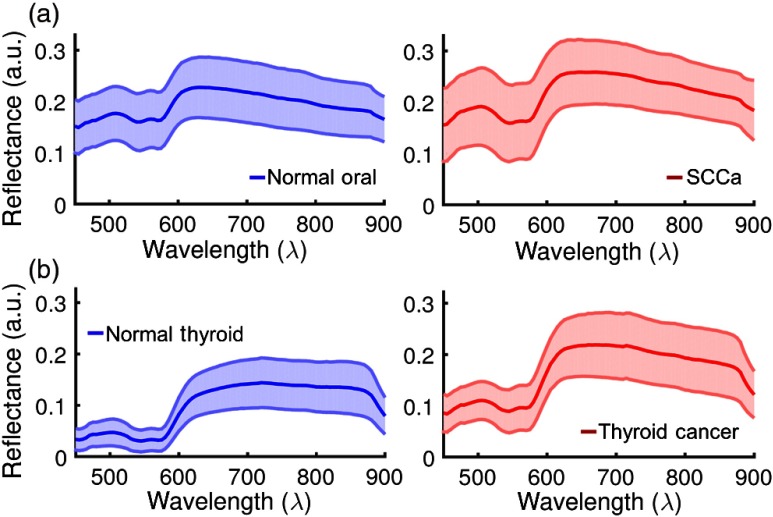
(a) Normalized reflectance curves for the average spectra, shown with standard deviation, of all 29 SCCa patients. (b) Normalized reflectance curves for the average spectra of all 21 thyroid patients.

**Fig. 2:**
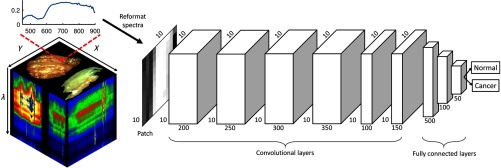
Flowchart of the data processing and deep learning architecture. The spectral signatures from 5×5 blocks extracted from the hypercube are reformatted into 10×10 spectral patches. The CNN trained on the spectral patches consisted of six convolutional layers (height, width, and filter numbers are shown) and three fully connected layers (number of neurons in the layer are shown).

After normalization and glare removal, pixels were averaged in 5×5, nonoverlapping neighborhoods in order to obtain average spectra.^6^ Therefore, each block contains a normalized and averaged reflectance-based spectral signature that has one grayscale intensity value for each of the 91 bands. Figure 1 shows the average spectral signature for each tissue type, which was constructed by averaging all blocks from all tissue samples of the corresponding tissue class. Next, a spectral patch is constructed from each block using the 91 reflectance values along with padding zeros and reformatting the spectral signature into a 10×10  pixel patch.^7^ As shown in Fig. 2, the spectral patches produced from all normal and cancer tissue samples are used for classification.

### 2.4 Convolutional Neural Network

A CNN was implemented using TensorFlow to classify the spectral patches as either normal or cancer tissue.^8^^–^^11^ The neural network architecture consisted of six convolutional layers and three fully connected layers. The number of filters in each convolutional layer and the number of neurons in each fully connected layer are shown in Fig. 2. The patch size used was 10×10, and the kernel size used for convolutions was 3×3. The output of each convolutional layer is 10×10×N, where N is the number of filters in the convolutional layer. The final layer, i.e., soft-max, generates a probability of the pixel belonging to either class. Neuron weights were initialized to 0.05 with a truncated normal distribution, and the learning rate is 0.01 with an adaptive gradient descent algorithm used for optimization. The CNN was trained for 25,000 steps, using a batch size of 250 and five epochs of data.

### 2.5 Validation

As class labels are required for both training and performance evaluation, a gold-standard is, therefore, necessary. After image acquisitions, histological, digitized images were obtained from the surface cross section of the fixed tissues. This histological image was used to outline a gold standard by a head-and-neck specialized pathologist (JVL). Using the gold standard, a binary mask is made for class labels of each pixel within the normal and tumor sample.

The CNN classification performance was evaluated using leave-one-patient-out external-validation to calculate the sensitivity, specificity, and accuracy.^6^ For example, the CNN was trained on 49 patients’ normal and cancer tissue data, after which the normal and cancer tissue data from the 50th patient was classified using the fully trained CNN. A total of 37 external-validations were performed using all patients with histologically confirmed normal and tumor tissue samples (see Table 1). Performance was evaluated every 5000 steps, and training was stopped once the best performance was achieved. The training time for one external-validation was at an average of 1.5 h and the testing time was ∼30  s.

**Table 1:** Results of average CNN performance on patient held-out external validation, values are % ± SD.

	All patients	SCCa trained on SCCa only	SCCa trained on both	Thyroid trained on thyroid only	Thyroid trained on both
Sensitivity	81±19	77±21	79±15	86±23	83±23
Specificity	78±20	78±19	67±20	93±9	92±9
Accuracy	80±14	77±16	74±14	90±10	88±11

To further investigate interpatient variability, the patients were separated according to their cancer type into two groups, i.e., SCCa of the upper aerodigestive tract sites and cancer of the thyroid. The SCCa group had 29 patients from whom 20 external-validations were performed, and the thyroid cancer group had 21 patients from whom 17 external-validations were performed, as shown in Table 1.

The cross-validation method of performance evaluation involves taking patient samples that are known to be of one class for the CNN training, and then classifies new tissue from that same patient for validation. This technique could augment the performance of the classification when a surgeon can provide a sample from the patient for training. This method provides the benchmarks for the proposed CNN approach (see Table 2). The spectral patches from all 50 patients were randomly divided into two, nonoverlapping groups, i.e., the training and testing datasets. Seventy-five percent of the spectral patches were used as the training dataset, and the remaining 25% comprised the testing dataset. The CNN was fully trained for 20,000 steps using the training dataset, and the performance was calculated using the testing dataset. The performance of the classifiers, SVM (Gaussian kernel, manual scale set to 3.5), kNN (k=10, squared inverse Euclidean distance), logistic regression (LR), complex decision tree classifier (DTC: Gini index with 100 splits), and linear discriminant analysis (LDA: diagonal convergence),^1^^,^^12^^–^^14^ all of which were implemented in MATLAB, was evaluated.

**Table 2:** Performance of CNN and other machine learning methods on the 75%/25% training/testing data cross validation, different regions from the same patients are used between groups.

Classifier	Sensitivity (%)	Specificity (%)	Accuracy (%)
CNN^a^	96.8	96.1	96.4
SVM	93.0	91.6	92.3
kNN	91.9	86.9	89.4
LR	81.4	82.2	81.8
DTC	85.8	72.6	79.3
LDA	66.1	68.7	67.4

a represents the proposed method.

## 3 Results

**Fig. 3:**
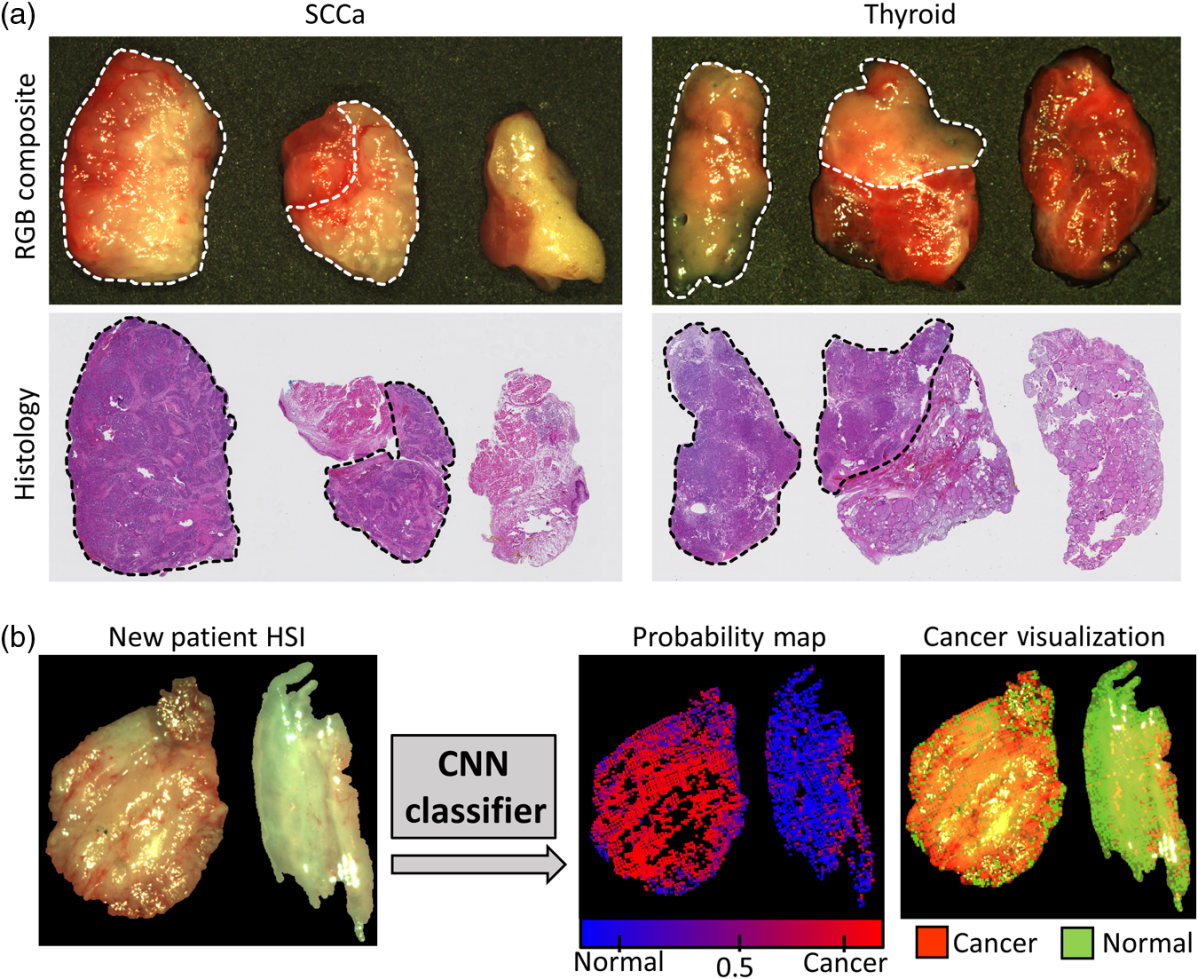
(a) Representative HSI-RGB composite and histological images from maxillary sinus SCCa (left) and thyroid (right) patients. The dotted line indicates the cancer margin. (b) Representative CNN classification results of a larynx SCCa patient.

The proposed CNN classifier can identify cancer and normal tissue with 81% sensitivity, 78% specificity, and 80% accuracy. See Table 1 for the complete results. A representative pseudocolor visualization of the results is provided in Fig. 3. The performance of both the SCCa and thyroid groups was decreased by augmenting the training group with normal and cancer samples of the other group. The SCCa group performed with 74% accuracy when trained on tissues from both the aerodigestive tract and the thyroid but achieved 77% accuracy when trained on aerodigestive tract tissue only. Likewise, the thyroid cancer group had 88% accuracy when trained on tissues from both the aerodigestive tract and the thyroid but performed with 90% accuracy when trained on thyroid tissue only. The large standard deviations are created by some patients classified with low accuracy and some being classified with near perfect accuracy.

The second method for performance evaluation, which simulates augmenting the tissue database with known patient sample data, had 97% sensitivity, 96% specificity, and 96% accuracy. This cross-validation method should be expected to have better performance than the external validation method because it trains and tests on different regions from the same patient and is mainly used for comparison of different machine learning techniques. Moreover, we can see that the proposed CNN classifier outperformed all of the evaluated machine learning algorithms, and the top scoring results are shown in Table 2.

## 4 Conclusion

Our experimental results show that the CNN has potential for use in the automatic labeling of cancer and normal tissue using hyperspectral images, which could be useful for intraoperative cancer detection. The proposed technique is fast and does not require any further postprocessing to enhance the results. Moreover, the 37-fold, leave-one-out external-validation shows that the classification technique is reliable and can be applied to new patient images. Further studies will involve incorporating more patient HSI data, comparing the effect of dimensionality reduction, and investigating more network structures and neuron initialization techniques to optimize classification performance and improve generalizability.
